# Missense mutation outside the forkhead domain of *FOXL2* causes a severe form of BPES type II

**Published:** 2012-01-26

**Authors:** Alireza Haghighi, Hannah Verdin, Hamidreza Haghighi-Kakhki, Niloofar Piri, Nasrollah Saleh Gohari, Elfride De Baere

**Affiliations:** 1 Wellcome Trust Centre for Human Genetics, University of Oxford, Oxford, UK; 2 Center for Medical Genetics, Ghent University Hospital, Ghent, Belgium; 3 Faculty of Medicine, Mashhad Azad University, Mashhad, Iran; 4 Department of Ophthalmology, Alavi Eye Hospital, Ardebil University of Medical Sciences, Ardebil, Iran; 5 Department of Genetics, Kerman University of Medical Sciences, Kerman, Iran

## Abstract

**Purpose:**

Blepharophimosis-ptosis-epicanthus inversus syndrome (BPES) is a developmental disease characterized by a complex eyelid malformation associated or not with premature ovarian failure (POF). BPES is essentially an autosomal dominant disease, due to mutations in the forkhead box L2 (*FOXL2*) gene, encoding a forkhead transcription factor. More than one hundred unique *FOXL2* mutations have been described in BPES in different populations, many of which are missense mutations in the forkhead domain. Here, we report on a very severe form of BPES resulting from a missense mutation outside the forkhead domain.

**Methods:**

A clinical and molecular genetic investigation was performed in affected and unaffected members of an Iranian family with BPES. The *FOXL2* coding region was sequenced in an index case. Targeted mutation testing was performed in 8 family members.

**Results:**

We have identified a heterozygous *FOXL2* missense mutation c.650C→G (p.Ser217Cys) co-segregating with disease in members of a three-generation family with BPES type II. Only few missense mutations have been reported outside the forkhead domain so far. They were all found in mild BPES, in line with in vitro studies demonstrating mostly normal localization and normal or increased transactivation properties of the mutant proteins. Unlike previous studies, affected members of the family studied here showed a severe BPES phenotype, with bilateral amblyopia due to uncorrected ptosis.

**Conclusions:**

This is the first study demonstrating a severe BPES phenotype resulting from a *FOXL2* missense mutation outside the forkhead domain, expanding our knowledge about the phenotypic consequences of missense mutations outside the forkhead domain in BPES.

## Introduction

Blepharophimosis, ptosis and epicanthus inversus syndrome (BPES; OMIM 110100) is a rare genetic condition basically inherited in an autosomal dominant fashion [1]. The global prevalence of BPES has been estimated to be ~1 in 50,000 [2]. Two types of BPES have been identified: BPES type I is a complex eyelid malformation associated with premature ovarian failure (POF), whereas in BPES type II, only the eyelid defect is present [3]. The major features of the eyelid malformation involve (1) narrowed horizontal aperture of the eyelids (blepharophimosis), (2) drooping of the upper eyelid (ptosis), (3) the presence of a fold of skin arising from the lower eyelid that runs inward and upward (epicanthus inversus), and (4) lateral displacement of the inner canthi (telecanthus) [2,3]. Decock et al. [4] reported that the lateral displacement of the inferior lacrimal puncta is an important anatomic hallmark of BPES, having applications in the clinical diagnosis and in the improvement of BPES surgery.

BPES is caused by mutations in the single-exon gene forkhead box L2 (*FOXL2*; OMIM 605597) that encodes a forkhead transcription factor [5,6]. The FOXL2 protein is a highly conserved protein of 376 amino acids containing a 110-amino-acid DNA-binding forkhead domain and a polyalanine tract that is conserved in mammals. The expression pattern of FOXL2 is compatible with the BPES phenotype, as expression studies in human, mouse, and goat demonstrated the presence of the nuclear protein in the mesenchyme of developing eyelids and in fetal and adult supporting granulosa cells but not in the oocytes [7]. It is the earliest known marker of ovarian differentiation in mammals. Moreover, *FOXL2* is strongly expressed in adult follicular cells, suggesting not only a role in ovarian somatic cell differentiation but also in adult female fertile life in follicular development and maintenance [7]. *FOXL2* expression has also been demonstrated in the developing and adult pituitary [5,8]. In addition, a wider expression domain is suggested by online resources (Gene Expression Omnibus; GEO).

Of all genetic defects identified in BPES, intragenic mutations represent the largest group (81%) [6,9]. Deletions encompassing *FOXL2* and located outside its transcription unit represent 12% and 5% of molecular defects respectively [10]. More than 100 unique FOXL2 mutations have been described in BPES. The largest group (44%) contains frameshift mutations. Following are the in-frame changes (33%, of which polyalanine expansions represent the largest group), the nonsense mutations (12%) and finally the missense mutations (11%). Several genotype-phenotype correlations emerged after the identification of the first *FOXL2* mutations. Initially, it was proposed that mutations predicted to result in proteins with truncation before the polyalanine tract might by associated with BPES type I, whereas polyalanine expansions might rather lead to BPES type II. For missense mutations and mutations leading to a truncated or extended protein containing an intact forkhead domain and polyalanine tract, no correlations could be made [6,11]. Functional studies investigating the consequences of *FOXL2* missense mutations have shed light on the molecular pathogenesis of BPES, and contributed to genotype-phenotype correlations [12-15]. From the first mutation studies it was hypothesized that these mutations were loss-of-function alleles leading to haploinsufficiency of FOXL2 [5,6]. This was supported by the observation that *FOXL2* deletions and intragenic mutations lead to the same phenotype [6]. This was not clear for missense mutations however.

In this study, we investigated the clinical presentation and molecular genetic basis of BPES in a three-generation family from Iranian origin. We demonstrated a severe phenotypic effect of a *FOXL2* missense mutation outside the forkhead domain.

## Methods

### Patients

**Figure 1:**
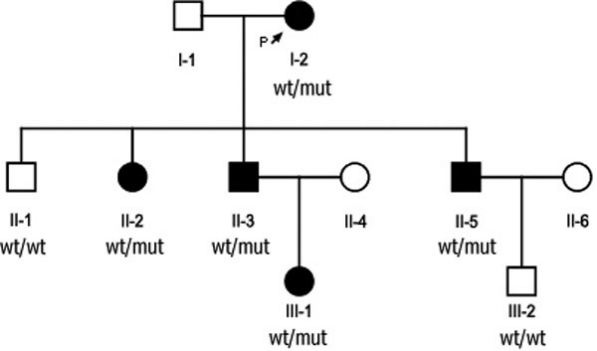
Pedigree of the BPES type II family. Affected individuals are indicated with filled symbols. Abbreviations: wt: wild type allele; mut: mutant allele.

Recruitment of the family was based on interviews, questionnaires, and clinical examination of affected and unaffected individuals by ophthalmologists and geneticists. An informed consent was obtained in compliance with the Helsinki Declaration. The pedigree of the family is shown in Figure 1. Best corrected visual acuity (BCVA) was measured in all patients by the Early Treatment Diabetic Retinopathy Study (ETDRS) chart.

### Mutation screening

Leukocyte genomic DNA was extracted from peripheral venous blood from nine subjects by use of standard methods. Amplification and subsequent sequencing of the entire coding region of *FOXL2* was performed as described in the index case [6]. Briefly, four primer sets were designed for the coding region of *FOXL2* (Table 1). PCR was conducted in 25 µl reactions containing 100 ng DNA, 10× PCRx Amplification Buffer (Invitrogen, Carlsbad, CA), 10× PCRx Enhancer Solution, 0.25 mmol/l of each dNTP, 25 mmol/l MgSO_4_, 1 unit PlatinumTaq DNA polymerase (Invitrogen) and 50 µmol/l of each oligodeoxynucleotide primer (IDT, Coralville, IA). Cycling conditions included one cycle of 94 °C for 4 min followed by 35 cycles of 94 °C for 20 s, 56 °C for 20 s, and 72 °C for 40 s, and one cycle of 72 °C for 10 min. Sequencing was performed with the BigDye Terminator v3.1 Cycle Sequencing Kit on a ABI 3730XL genetic Analyzer, according to the manufacturer’s instructions (Applied Biosystems, Carlsbad, CA). Segregation analysis was performed in eight affected and unaffected family members. Mutation nomenclature is based on GenBank entry AF301906.1, with +1 corresponding to the A of the translation initiation codon ATG in the cDNA nomenclature, according to the Human Genome Variation Society (HGVS) nomenclature guidelines.

**Table 1:** PCR primers and conditions.

**Set**	**Forward sequence (5’→3’)**	**Melting temperature (°C)**	**Reverse sequence (5’→3’)**	**Melting temperature (°C)**	**PCR product size (bp)**
***FOXL2* primers**					
1	ctaggggaaggggaaggag	60.0	gttgtggcggatgctatttt	60.0	500
2	cgaagttcccgttctacgag	59.9	gcatagggcatgggtgag	60.0	391
3	gacggctacggctacctg	59.4	ccaggccattgtacgagttc	60.5	283
4	ccggcgtagtgaactcgta	59.9	aaagcgaaaaagcacagagg	59.6	486
**5’ UTR primer**					
1	ctccttgactgtgcgac	53.1	aaagtgacttggagatgaact	53.0	594
**3’ UTR primers**					
1	gagcgacagaaataaagaagtcc	58.6	ttcaaacctcctgcttctcc	59.4	271
2	cgggtttcacatttctcctt	59.0	ggaagtattgtggccttgga	59.9	366
3	gattttcatatttggatttagcaaac	58.6	gccggacaggactgatgg	62.7	400
4	cggagcaaacacacgtattg	60.2	agggtccctctgtgcttttt	60.1	390
5	gagcgacaggagcttaggaa	59.7	gccatgatgcattgctctta	59.8	247

### Screening of the untranslated regions (UTRs)

To detect regulatory mutations, sequencing of the 5′ and 3′ untranslated region (UTR) of *FOXL2* was performed in the index patient and three affected family members. One and five primer sets were designed for the 5’ UTR and 3’ UTR, respectively (Table 1). PCR for primer set 1 and 2 from the 3’ UTR was conducted in 30 μl reactions containing 100 ng DNA, 10× PCR Buffer (Invitrogen), 0.25 mmol/l of each dNTP, 50 mmol/l MgCl_2_, 1 unit PlatinumTaq DNA polymerase (Invitrogen) and 50 µmol/l of each oligodeoxynucleotide primer (IDT). Cycling conditions included one cycle of 94 °C for 4 min followed by 35 cycles of 94 °C for 20 s, 55 °C for 20 s, and 72 °C for 40 s, and one cycle of 72 °C for 10 min. For the other primer sets, PCR was conducted in 30 μl reactions containing 100 ng DNA, 10× PCRx Amplification Buffer (Invitrogen), 10× PCRx Enhancer Solution, 0.25 mmol/l of each dNTP, 50 mmol/l MgSO_4_, 1 unit PlatinumTaq DNA polymerase (Invitrogen) and 50 µmol/l of each oligodeoxynucleotide primer (IDT). Touchdown cycling conditions included one cycle of 94 °C for 5 min followed by 10 cycles of 94 °C for 30 s, 65 °C (decreasing 1 °C in every cycle) for 15 s, and 72 °C for 60 s followed by 35 cycles of 94 °C for 45 s, 55 °C for 45 s, and 72 °C for 90 s and one cycle of 72 °C for 10 min. The UTRs of *FOXL2,* were examined by direct Sanger sequencing of PCR products on an ABI 3730XL Genetic Analyzer, according to the manufacturer’s instructions (Applied Biosystems).

### Quantitative PCR (qPCR) in the *FOXL2* region

To detect copy number variations in the *FOXL2* region, quantitative PCR (qPCR) of the shortest region of overlap (SRO) was performed as described [16]. In short, 3 qPCR amplicons located in the SRO were analyzed for the presence of copy number variants in two affected family members. qPCR was performed using the qPCR core kit for SYBR Green I (Eurogentec, Serzing, Belgium) on the LightCycler 480 Instrument II (Roche Applied Science, Penzberg, Germany). Data-analysis was performed with the commercially available qBasePlus software [17]. Two reference genes were used for normalization of the relative quantities and two positives controls with known copy number were used as a reference to calculate the copy numbers [18].

## Results

### Clinical evaluation

We identified a three-generation Iranian family with multiple members having BPES. The 60 years old female proband (I-2) was the first member known to be affected by BPES. She was born at term to non-consanguineous healthy parents after an uneventful pregnancy. Her other five siblings were normal. Six affected members in this family span three generations.

**Figure 2:**
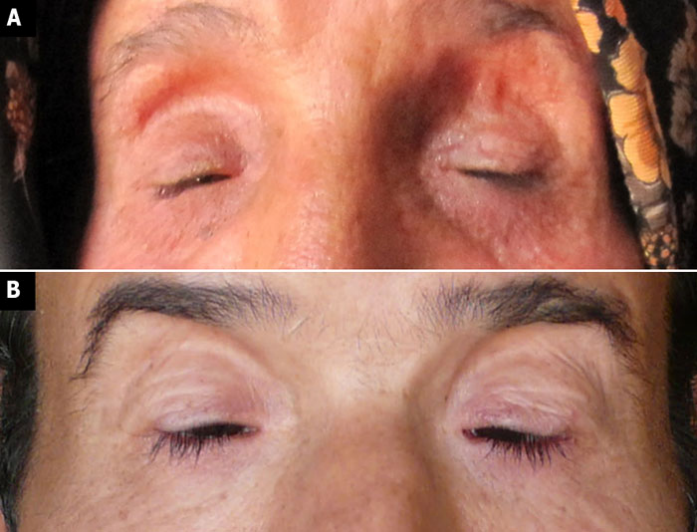
BPES features in affected individuals. A severe form of BPES can be appreciated in proband I-2 (**A**) and II-5 (**B**), with amblyopia due to uncorrected, severe ptosis.

The proband presented with severe BPES (Figure 2A). She did not undergo any oculoplastic surgery. An ophthalmologic exam revealed that she had high myopia, bilateral amblyopia, and best corrected visual acuity (BCVA) of 20/100 in both eyes (cyclorefraction: OD: −11.25–3.00x77°; OS: −10.00–2.75x85°). Slit lamp evaluation revealed a normal anterior segment apart from mild nuclear sclerosis. Fundoscopy demonstrated high myopic changes.

Her son (II-5), 34 years old, presented with high myopia and significant cataract in both eyes. He had horizontal nystagmus due to deep amblyopia of both eyes and right esotropia. BCVA in his right and left eyes was counting fingers at 1.5 m and at 2 m respectively. Fundoscopy showed high myopic changes similar to his mother. He reported an unsuccessful repair surgery at the age of 18 (Figure 2B).

A granddaughter (III-1) was 6 years old. She had a successful eyelid blepharoplasty (VY-plasty) when she was 6 months. BCVA was 20/20 and 20/30 in her right and left eye respectively (cyclorefraction: OD: +1.50–2.00x100°; OS: +1.00–1.25x70°). She had an otherwise normal ocular examination.

**Figure 3:**
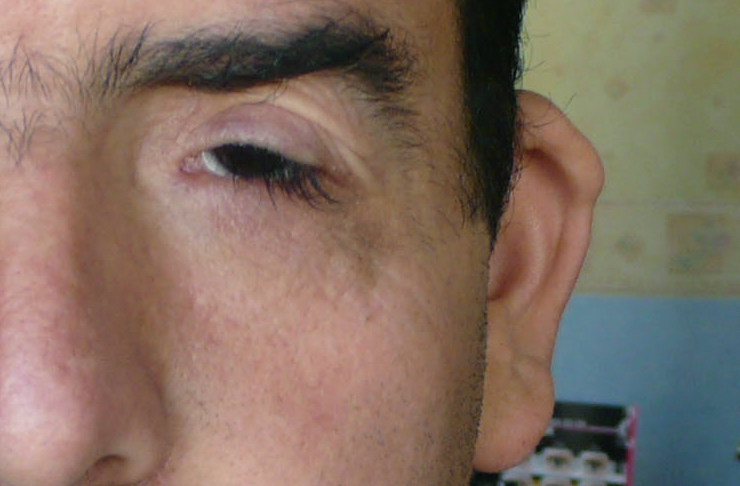
Example of abnormal lop ear (left side) in proband II-5.

Apart from malformed lop ears in one of the patients (II-5; Figure 3), no extra-ocular abnormalities were found in the other members. None of the female probands had oligomenorrhea or signs of ovarian dysfunction and reproductive problems.

### Genetic study

**Figure 4:**
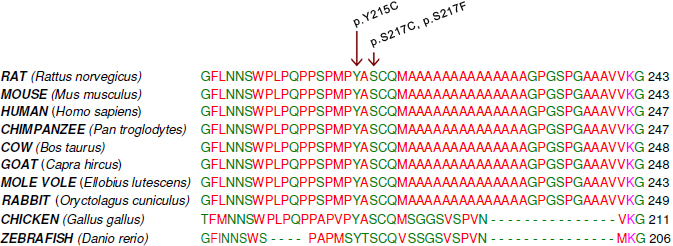
Multiple sequence alignment of the FOXL2 protein region flanking residue Ser217. ClustalW analysis demonstrates that amino acid serine at position 217 is well conserved in orthologs. Known missense mutations reported in BPES downstream of the forkhead domain and upstream of the polyalanine tract are indicated by arrows.

Sequencing of the coding exon of *FOXL2* in the proband (I-2) revealed a heterozygous missense mutation c.650C→G (p.Ser217Cys). Targeted testing of this mutation showed presence of this mutation in all affected family members tested in a heterozygous state, and absence in the unaffected members (Figure 1). Mutation p.Ser217Cys is a known missense mutation previously described in an Indian pedigree [13] (Table 2). A different missense mutation in the same residue p.Ser217Phe has been described previously in two siblings with mild BPES [12] (Table 2). The Ser217 residue is located outside the forkhead domain and is conserved throughout evolution (Figure 4). Polyphen and SIFT predictions suggest an effect on protein function (Table 2). The Grantham distance between the Ser and Cys residue is 112. Taken together, the p.Ser217Cys mutation can be considered as the causal mutation in this family.

**Table 2:** Natural *FOXL2* missense mutations outside the forkhead domain reported to date in BPES: clinical and molecular genetic data, in silico predictions and in vitro studies.

***FOXL2* mutation (nucleotide level)**	**FOXL2 mutation (protein level)**	**Phenotype**	**Clinical data**	**In silico predictions (conservation, Grantham distance, Polyphen, SIFT)**	**Subcellular distribution of mutant FOXL2-GFP**	**Transactivation properties of mutant FOXL2-GFP**
c.644A>G	p.Tyr215Cys (p.Y215C) [19].	BPES familial	Mild BPES (no epicanthus inversus, no broad and low nasal bridge, normal visual acuity) in five generation Indian family. BPES type could not be assessed. Co-segregation of mutation with disease.	Conservation: high up to Opossum (considering 11 species). Grantham distance: 194. Polyphen: probably damaging. SIFT: affect protein function (deleterious).	Intranuclear aggregation (p<0.001 in comparison with wild type protein) [14].	Similar transactivation capacities compared to wild type protein (4xFLRE-luc and SIRT1-luc constructs) [14].
c.650C>T	p.Ser217Phe (p.S217F) [12].	BPES familial	Mild BPES. Co-segregation of mutation with disease.	Conservation: moderate (considering 11 species). Grantham distance: 155. Polyphen: possibly damaging. SIFT: affect protein function (deleterious)	No impairment compared to wild type [12].	Increased transactivation compared to wild type using DK3 promotor [12]. Classified as type II mutations using 4xFLRE-luc and SIRT1-luc constructs [14].
c.650C>G	p.Ser217Cys (p.S217C) [13].	BPES familial	Mild BPES	Conservation: moderate (considering 11 species). Grantham distance: 112. Polyphen: possibly damaging. SIFT: tolerated	No impairment compared to wild type [13].	No change compared to wild type [13]. Classified as type II mutations using 4xFLRE-luc and SIRT1-luc constructs [14].
c.650C>G	p.Ser217Cys (p.S217C). This study.	BPES familial (type 2)	Severe BPES. Co-segregation of mutation with disease.	See above	See above	See above

Sequencing of the UTRs of *FOXL2* in the proband and three affected family members did not reveal a sequence variant. Copy number analysis in two affected family members using qPCR of the SRO containing a long non-coding RNA and several conserved non-coding sequences (CNCs) which interact with the *FOXL2* promotor [16], did not reveal copy number variations (data not shown).

## Discussion

A heterozygous missense mutation of *FOXL2*, p.Ser217Cys, was found to underly the phenotype here. Unlike most missense mutations, this mutation is located outside the forkhead domain between the forkhead domain and the polyalanine tract. All missense mutations outside the forkhead domain found in BPES are summarized in Table 2, with their phenotypic consequences, in silico predictions and in vitro assays, if any. In this study, affected patients presented with a severe form of BPES, leading to amblyopia and poor BCVA in individuals who did not undergo oculoplastic surgery. In females no signs of ovarian dysfunction were present, suggesting occurrence of type II BPES. This mutation was previously reported by Nallathambi et al. [13] in an Indian BPES family, with a mild eyelid phenotype. A different mutation affecting the same residue p.Ser217Phe was found in a Belgian BPES family, in which a father and two pre-pubertal daughters displayed a similarly very mild BPES phenotype [12]. Apart from the ocular findings, one of the siblings presented with alopecia areata, and the other one with growth hormone deficiency [12]. In addition, Kumar et al. [19] reported on missense mutation p.Tyr215Cys in an Indian family, in which all affected individuals exhibited mild to typical BPES with a normal visual acuity and normal ocular examination including mobility. All the patients except individual III-2 had chin elevation and telecanthus. Levator function was decreased in all the patients [19]. From these previous studies was concluded that missense mutations outside the forkhead domain might lead to a rather mild BPES phenotype. In comparison with these previous mild cases however, the BPES phenotype of the affected individuals reported here is very severe. In those members who did not undergo successful oculoplastic surgery (I-2 and II-5), this led to bilateral amblyopia and poor BVCA.

Insights into the molecular effects of *FOXL2* missense mutations contributing to genotype-phenotype correlations resulted from in vitro studies. First, the missense change p.Ser217Phe was shown to have no effect on subcellular localization of the FOXL2 protein and to increase its transactivation capacity on the *DK3* promoter, suggesting hypermorphism [12]. This might be in agreement with previous observations in *FOXC1*-related phenotypes, in which haploinsufficiency of FOXC1 and hypermorphism (due to gene duplications) lead to similar but not identical anterior segment phenotypes [20]. Reasoning the same way for FOXL2, this might explain why hypermorphic mutations such as p.Ser217Phe would give rise to a somewhat different, mild BPES phenotype. An equally mild BPES phenotype resulted from a different mutation in the same residue (p.Ser217Cys) of which neither localization nor transactivation was impaired [12,14]. As said, we identified the p.Ser217Cys mutation in a severe form of BPES here. As this severe ocular phenotype was observed in all affected individuals of different generations, this might be attributed for instance to a *cis*-effect of regulatory variants within or outside the transcription unit modulating the expression of mutant *FOXL2*. Notably, as the origins of the families described by Nallathambi et al. [13] and here are different (i.e., Indian versus Iranian, respectively), different haplotypes and regulatory contexts might be expected. As we could not substantiate this hypothesis so far by means of sequencing of the UTRs and by copy number screening of previously described SRO of regulatory deletions this remains speculative. However, several alternative possibilities, such as subtle sequence changes in regulatory elements, or promoter variations cannot be ruled out.

In addition, the phenotypic effect seems to be tissue-specific, as no ovarian involvement was observed in any of the affected females here. Indeed, this is in line with the study by Dipietromaria et al. [14], in which a classification tool was developed for *FOXL2* intragenic (missense and other) mutations, correlating the transcriptional activity of *FOXL2* mutations on two different reporter promoters and the BPES type [14]. Following this classification system, both p.Ser217Cys and p.Ser217Phe can be categorized as type II BPES mutations.

In conclusion, this study has expanded our knowledge about the phenotypic consequences of missense mutations outside the forkhead domain of *FOXL2* by the identification of p.Ser217Cys, for the first time, in a very severe form of BPES in a family of Iranian descent.

## References

[1] Kohn R, Romano PE (1971). Blepharoptosis, blepharophimosis, epicanthus inversus, and telecanthus–a syndrome with no name.. Am J Ophthalmol.

[2] Oley C, Baraitser M (1988). Blepharophimosis, ptosis, epicanthus inversus syndrome (BPES syndrome).. J Med Genet.

[3] Zlotogora J, Sagi M, Cohen T (1983). The blepharophimosis, ptosis, and epicanthus inversus syndrome: delineation of two types.. Am J Hum Genet.

[4] Decock CE, Claerhout I, Leroy BP, Kesteleyn P, Shah AD, De Baere E (2011). Correction of the lower eyelid malpositioning in the blepharophimosis-ptosis-epicanthus inversus syndrome.. Ophthal Plast Reconstr Surg.

[5] Crisponi L, Deiana M, Loi A, Chiappe F, Uda M, Amati P, Bisceglia L, Zelante L, Nagaraja R, Porcu S, Ristaldi MS, Marzella R, Rocchi M, Nicolino M, Lienhardt-Roussie A, Nivelon A, Verloes A, Schlessinger D, Gasparini P, Bonneau D, Cao A, Pilia G (2001). The putative forkhead transcription factor FOXL2 is mutated in blepharophimosis/ptosis/epicanthus inversus syndrome.. Nat Genet.

[6] De Baere E, Dixon MJ, Small KW, Jabs EW, Leroy BP, Devriendt K, Gillerot Y, Mortier G, Meire F, Van Maldergem L, Courtens W, Hjalgrim H, Huang S, Liebaers I, Van Regemorter N, Touraine P, Praphanphoj V, Verloes A, Udar N, Yellore V, Chalukya M, Yelchits S, De Paepe A, Kuttenn F, Fellous M, Veitia R, Messiaen L (2001). Spectrum of FOXL2 gene mutations in blepharophimosis-ptosis-epicanthus inversus (BPES) families demonstrates a genotype–phenotype correlation.. Hum Mol Genet.

[7] Cocquet J, De Baere E, Gareil M, Pannetier M, Xia X, Fellous M, Veitia RA (2003). Structure, evolution and expression of the FOXL2 transcription unit.. Cytogenet Genome Res.

[8] Treier M, Gleiberman AS, O'Connell SM, Szeto DP, McMahon JA, McMahon AP, Rosenfeld MG (1998). Multistep signaling requirements for pituitary organogenesis in vivo.. Genes Dev.

[9] Beysen D, De Paepe A, De Baere E (2009). FOXL2 mutations and genomic rearrangements in BPES.. Hum Mutat.

[10] Beysen D, Raes J, Leroy BP, Lucassen A, Yates JR, Clayton-Smith J, Ilyina H, Brooks SS, Christin-Maitre S, Fellous M, Fryns JP, Kim JR, Lapunzina P, Lemyre E, Meire F, Messiaen LM, Oley C, Splitt M, Thomson J, Van de Peer Y, Veitia RA, De Paepe A, De Baere E (2005). Deletions involving long-range conserved nongenic sequences upstream and downstream of FOXL2 as a novel disease-causing mechanism in blepharophimosis syndrome.. Am J Hum Genet.

[11] De Baere E, Beysen D, Oley C, Lorenz B, Cocquet J, De Sutter P, Devriendt K, Dixon M, Fellous M, Fryns JP, Garza A, Jonsrud C, Koivisto PA, Krause A, Leroy BP, Meire F, Plomp A, Van Maldergem L, De Paepe A, Veitia R, Messiaen L (2003). FOXL2 and BPES: mutational hotspots, phenotypic variability, and revision of the genotype-phenotype correlation.. Am J Hum Genet.

[12] Beysen D, Moumne L, Veitia R, Peters H, Leroy BP, De Paepe A, De Baere E (2008). Missense mutations in the forkhead domain of FOXL2 lead to subcellular mislocalization, protein aggregation and impaired transactivation.. Hum Mol Genet.

[13] Nallathambi J, Laissue P, Batista F, Benayoun BA, Lesaffre C, Moumne L, Pandaranayaka PE, Usha K, Krishnaswamy S, Sundaresan P, Veitia RA (2008). Differential functional effects of novel mutations of the transcription factor FOXL2 in BPES patients.. Hum Mutat.

[14] Dipietromaria A, Benayoun BA, Todeschini AL, Rivals I, Bazin C, Veitia RA (2009). Towards a functional classification of pathogenic FOXL2 mutations using transactivation reporter systems.. Hum Mol Genet.

[15] Todeschini AL, Dipietromaria A, L'Hote D, Boucham FZ, Georges AB, Pandaranayaka PJ, Krishnaswamy S, Rivals I, Bazin C, Veitia RA (2011). Mutational probing of the forkhead domain of the transcription factor FOXL2 provides insights into the pathogenicity of naturally occurring mutations.. Hum Mol Genet.

[16] D'haene B, Attanasio C, Beysen D, Dostie J, Lemire E, Bouchard P, Field M, Jones K, Lorenz B, Menten B, Buysse K, Pattyn F, Friedli M, Ucla C, Rossier C, Wyss C, Speleman F, De Paepe A, Dekker J, Antonarakis SE, De Baere E (2009). Disease-causing 7.4 kb cis-regulatory deletion disrupting conserved non-coding sequences and their interaction with the FOXL2 promotor: implications for mutation screening.. PLoS Genet.

[17] Hellemans J, Mortier G, De Paepe A, Speleman F, Vandesompele J (2007). qBase relative quantification framework and software for management and automated analysis of real-time quantitative PCR data.. Genome Biol.

[18] D'haene B, Vandesompele J, Hellemans J (2010). Accurate and objective copy number profiling using real-time quantitative PCR.. Methods.

[19] Kumar A, Babu M, Raghunath A, Venkatesh CP (2004). Genetic analysis of a five generation Indian family with BPES: a novel missense mutation (p.Y215C).. Mol Vis.

[20] Lehmann OJ, Sowden JC, Carlsson P, Jordan T, Bhattacharya SS (2003). Fox's in development and disease.. Trends Genet.

